# Arsenic exposure in Indo Gangetic plains of Bihar causing increased cancer risk

**DOI:** 10.1038/s41598-021-81579-9

**Published:** 2021-01-27

**Authors:** Arun Kumar, Mohammad Ali, Ranjit Kumar, Mukesh Kumar, Prity Sagar, Ritu Kumari Pandey, Vivek Akhouri, Vikas Kumar, Gautam Anand, Pintoo Kumar Niraj, Rita Rani, Santosh Kumar, Dhruv Kumar, Akhouri Bishwapriya, Ashok Kumar Ghosh

**Affiliations:** 1 Mahavir Cancer Sansthan and Research Centre, Patna, Bihar 801505 India; 2 Department of Animal Sciences, Central University of Himachal Pradesh, Kangra, Himachal Pradesh India; 3 Amity Institute of Molecular Medicine and Stem Cell Research, Amity University Uttar Pradesh, Noida, India; 4 Department of Applied Geoscience and Engineering, Delft University of Technology, Delft, The Netherlands; 5 Geological Survey of India, Patna, Bihar India

**Keywords:** Cancer epidemiology, Cancer, Environmental sciences, Diseases, Oncology, Risk factors

## Abstract

Reportedly, 300 million people worldwide are affected by the consumption of arsenic contaminated groundwater. India prominently figures amongst them and the state of Bihar has shown an upsurge in cases affected by arsenic poisoning. Escalated arsenic content in blood, leaves 1 in every 100 human being highly vulnerable to being affected by the disease. Uncontrolled intake may lead to skin, kidney, liver, bladder, or lung related cancer but even indirect forms of cancer are showing up on a regular basis with abnormal arsenic levels as the probable cause. But despite the apparent relation, the etiology has not been understood clearly. Blood samples of 2000 confirmed cancer patients were collected from pathology department of our institute. For cross-sectional design, 200 blood samples of subjects free from cancer from arsenic free pockets of Patna urban agglomeration, were collected. Blood arsenic levels in carcinoma patients as compared to sarcomas, lymphomas and leukemia were found to be higher. The geospatial map correlates the blood arsenic with cancer types and the demographic area of Gangetic plains. Most of the cancer patients with high blood arsenic concentration were from the districts near the river Ganges. The raised blood arsenic concentration in the 2000 cancer patients strongly correlates the relationship of arsenic with cancer especially the carcinoma type which is more vulnerable. The average arsenic concentration in blood of the cancer patients in the Gangetic plains denotes the significant role of arsenic which is present in endemic proportions. Thus, the study significantly correlates and advocates a strong relation of the deleterious element with the disease. It also underlines the need to address the problem by deciphering the root cause of the elevated cancer incidences in the Gangetic basin of Bihar and its association with arsenic poisoning.

## Introduction

An estimated 300 million people worldwide are affected with arsenic poisoning leading to health hazards^1,2^. The contamination of groundwater with arsenic occurs either through anthropogenic or geogenic sources. People residing in different countries are exposed to increased doses of arsenic via consumption of arsenic-rich groundwater^3^. Worldwide, major arsenic hotspots have been identified in Taiwan, Chile, Mexico, China, Bangladesh, India and Argentina. Other incidents involving smaller population groups have been reported in Poland, Hungary, Japan, Canada and USA^4–11^.

Bihar, a state in Eastern India, located in Ganga-Meghna-Brahmaputra (GMB) basin faces problems of arsenic contamination in groundwater. Groundwater is the main source of drinking water which caters more than 80 per cent of drinking source in rural Bihar, hence the size of population exposed to adverse effects of arsenic is very high. The other sources of drinking water such as dug well, pond, surface water like lakes and rivers have lesser or no incidence of arsenic contamination, but these sources are not commonly utilized for the drinking purpose. Before 1980s, the open well water as ground water source were considered safe for drinking water but in recent years, due to increased anthropogenic activities and geogenic reasons, the arsenic contamination in the Gangetic plains has increased many folds. Out of 38 districts of the state of Bihar, 18 districts have been reported high arsenic contamination in groundwater^12^. It is estimated that more than 10 million people in Bihar are drinking water with arsenic concentration greater than the WHO/BIS permissible limit of 10 μg/L^13^. According to Ministry of Water Resources, Government of India, 1600 habitations from 67 blocks of 18 districts of the states are severely affected with arsenic poisoning. This has caused threat to an estimated 50 million population of the state out of which 13.85 million people are drinking arsenic contaminated water above 10 μg/L^14^. Hence, the existence of arsenic menace among the population is presently more than the estimated survey. In Bihar, the problem of arsenic poisoning in ground water was reported for the first time in Simaria Ojhapatti village of Bhojpur district. The exposed population was so severely affected, that most of the village people evacuated their households. The subjects exhibited typical symptoms of arsenicosis along with other internal diseases as well^15^. In the recent reports, it has been found that the districts of Bihar like Buxar, Bhojpur, Patna, Saran, Vaishali, Samastipur, Begusarai, Khagaria, Munger, Bhagalpur etc. lying close to the banks of Ganga river are severely affected by arsenic^13,16^. The use of arsenic contaminated drinking water is the major cause for skin, lung, bladder, kidney cancer as well as other adverse health effects such as skin manifestations, gastrointestinal disorders, neurological effects, hormone disruption and infertility, posing a global health concern^17–20^. Basically, the arsenic enters the body through drinking and passes through the gastrointestinal tract and reaches the blood which reaches the vital organs of the body and causes organ toxicity which in turn disrupts the metabolic function of the body causing disease in them^21–25^.

According to Globocan 2018, 18.0 million new cancer cases, 9.5 million death and 43.8 million relapses (within 5 year of survival) was reported^26^. According to the national data of National Cancer Registry Programme of the Indian Council of Medical Research (ICMR), India has reported 3.9 million cancer cases in 2016. The worst cancer affected states were Uttar Pradesh with 674,386 cases, followed by Maharashtra with 364,997 and Bihar with 359,228 while in south India, Tamil Nadu recorded 222,748 cases, Karnataka 202,156, Andhra Pradesh 159,696, Telangana 115,333 and Kerala 115,511 cases of cancer^27^.

The increased incidences of cancer in the state of Bihar has been a major challenge for the Government. The etiology of cancer incidences in this area has not been revealed properly. Hence, the present study is an approach to decipher the root cause of the cancer incidences in the Gangetic basin of Bihar and its association with arsenic.

## Materials and methods

### Location

The study was conducted at Mahavir Cancer Sansthan and Research Centre, Patna, Bihar. Altogether, 2000 cancer patients were identified and their blood samples were collected for the study. For the cross-sectional design, 200 blood samples of subjects free of cancer from arsenic free pockets of Patna urban agglomeration, Bihar were also collected as control.

### Selection of subjects for the study

- Cancer patients: In our cancer institute, approximately 15,000 confirm cancer cases are reported annually. For the present study, 2000 cancer confirm patients were randomly selected from the year 2017 to 2019. The selection of the patients was carried out randomly in the OPD of the institutes. For the diagnosis of the disease, various tests were carried out and after the confirmation of malignancy, they were recommended for the present study and their blood samples were collected.
- Control subjects: For the cross-sectional study, 200 subjects of urban Patna district of Bihar were selected as the control subjects. These subjects were from non- arsenic hit area of the district. These control subjects were taken in the study to compare the blood arsenic concentration between a normal subject versus cancer patients.

### Blood collection from the control subjects and cancer patients:

In the collection procedure, 5 ml of blood by volume was taken from the peripheral vein of the arm using disposable syringes and transferred to heparinised vaccutainer as per the guidelines of IUPAC^28^.

After the collection, all the blood samples were double digested using concentrated HNO_3_ on hot plate under fume hood and estimated as per the protocol of (NIOSH)^29^ through Graphite Furnace Atomic Absorption Spectrophotometer (Pinnacle 900T, Perkin Elmer, Singapore).

The patient based epidemiological data like patient’s age, gender, demographic area, cancer disease type, cancer stage etc. were collected from the patient files in the record room of MCSRC.

### GIS analysis and geo spatial mapping

The data of arsenic concentration in blood samples of the subjects were taken as input in Arc-GIS 10 software for spatial analysis, correlation, exposure rate and to visualize a synoptic view of the district-wise exposure rate. Concentrations of arsenic in blood data of the cancer patients were analysed with generation of statistical data in form of map and categorization therein. The arsenic background status map of Bihar was used for visualizing the exposure rate. All the layers were analysed using ArcGIS environment. The final output was generated as a thematic map. The software used in the map layer generation is ArcMap10.5.1, ESRI, ArcGIS Desktop 10.5.1 licensed at TU Delft Faculty of Civil Engineering and Geosciences. All the shapefiles were created in the ArcGIS environment for which base map was extracted from OpenStreetMap data downloaded from the link "http://download.geofabrik.de/asia/india.html". (OpenStreetMap contributors. (2017). Planet dump retrieved from https://planet.osm.org, https://www.openstreetmap.org).

The data was reported to the Mahavir Cancer Sansthan and Research Centre which brought to light significant findings in regards to the study.

### Statistical analysis

Data were analyzed with statistical software (Graph Pad Prism 5) and values were expressed as mean ± SEM. Differences between the groups were statistically analyzed by one-way analysis of variance (ANOVA) using the Dunnett’s test. The scattered graphs were plotted through another statistical software SPSS-16.0 using linear regression analysis model as earlier used^30^.

### Ethical approval

Ethical approval was obtained from the Institutional Ethics Committee (IEC) of Mahavir Cancer Sansthan and Research Centre with IEC No. MCS/Research/2015-16/2716, dated 08/01/2016.

## Results

The present study shows significant epidemiological information of 2000 cancer patients and 200 control subjects. The factors of age, gender, cancer type, blood arsenic concentration in blood of cancer patients and control subjects were given cognizance. Correlation coefficients of arsenic in blood and cancer subjects age, geospatial distribution of cancer patients and cancer type details were accounted.

**Figure 1:**
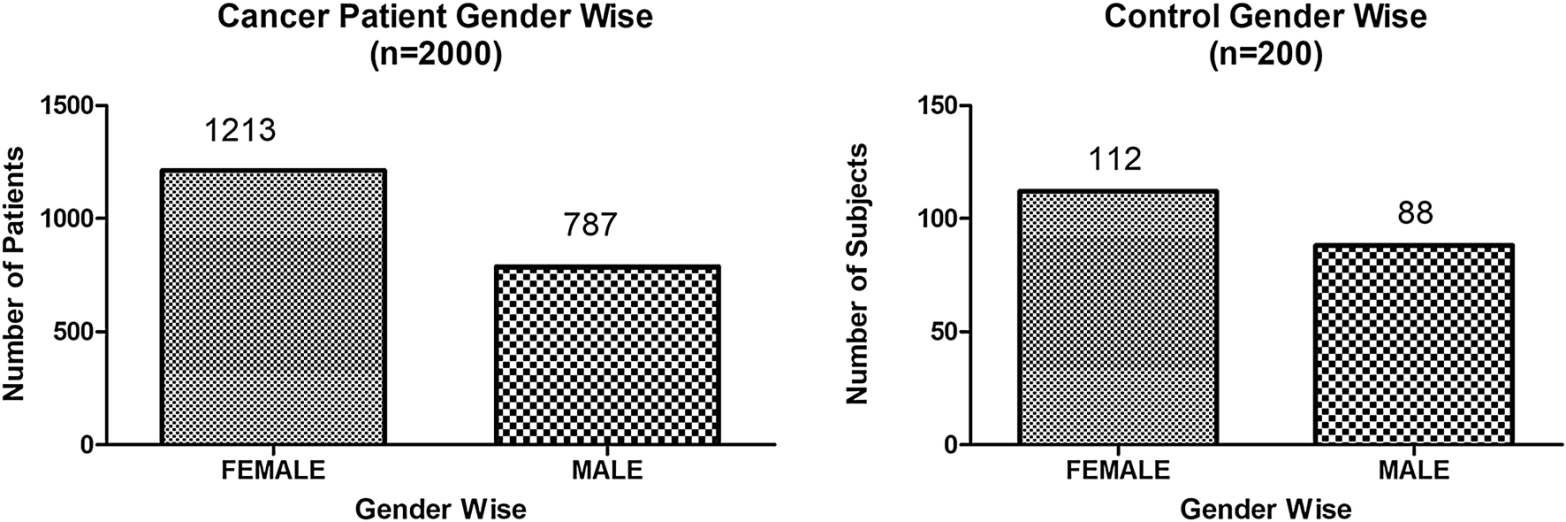
Graph showing number of patients (gender wise) in cancer patients and control subjects (ANOVA-Dunnett’s Test, P < 0.05).

**Figure 2:**
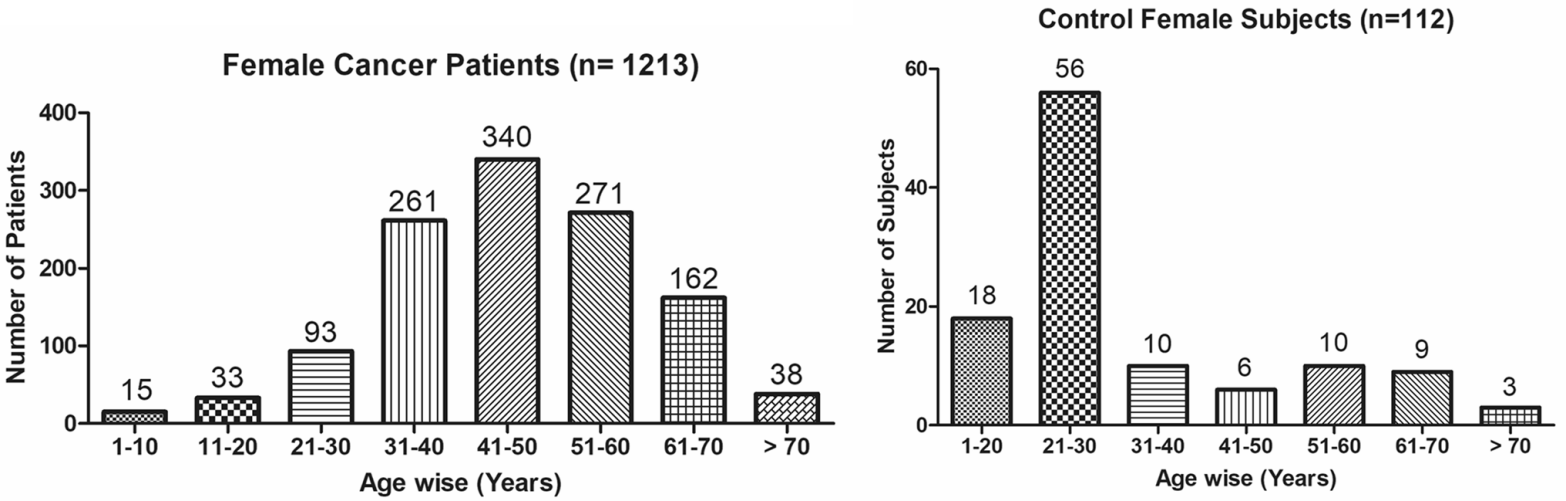
Graph showing age wise distribution of female cancer patients and control female subjects. (ANOVA-Dunnett’s Test, P < 0.05).

**Figure 3:**
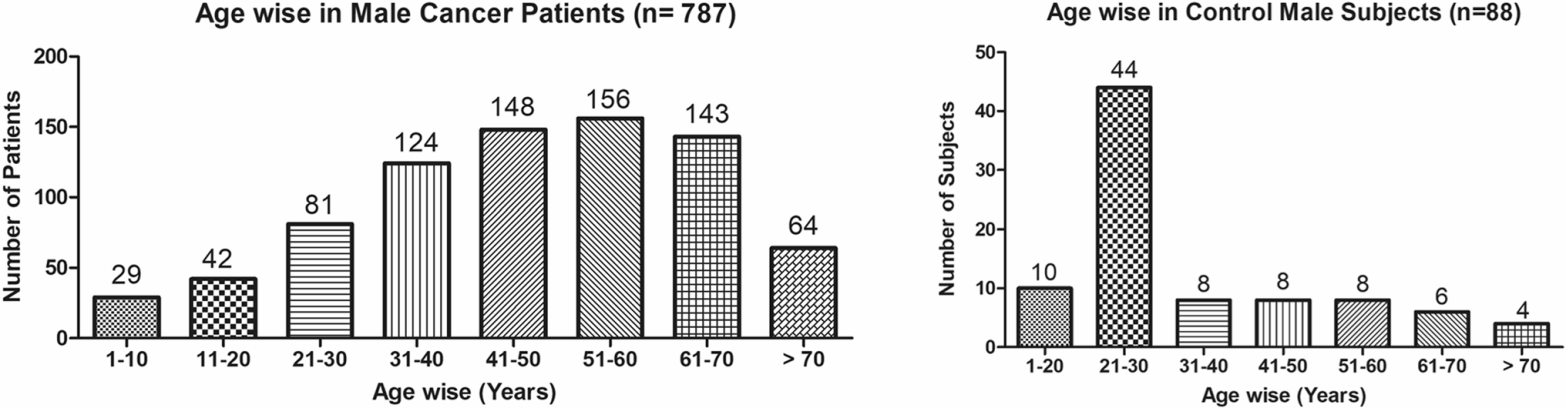
Graph showing age wise distribution of male cancer patients (ANOVA-Dunnett’s Test, P < 0.05).

**Figure 4:**
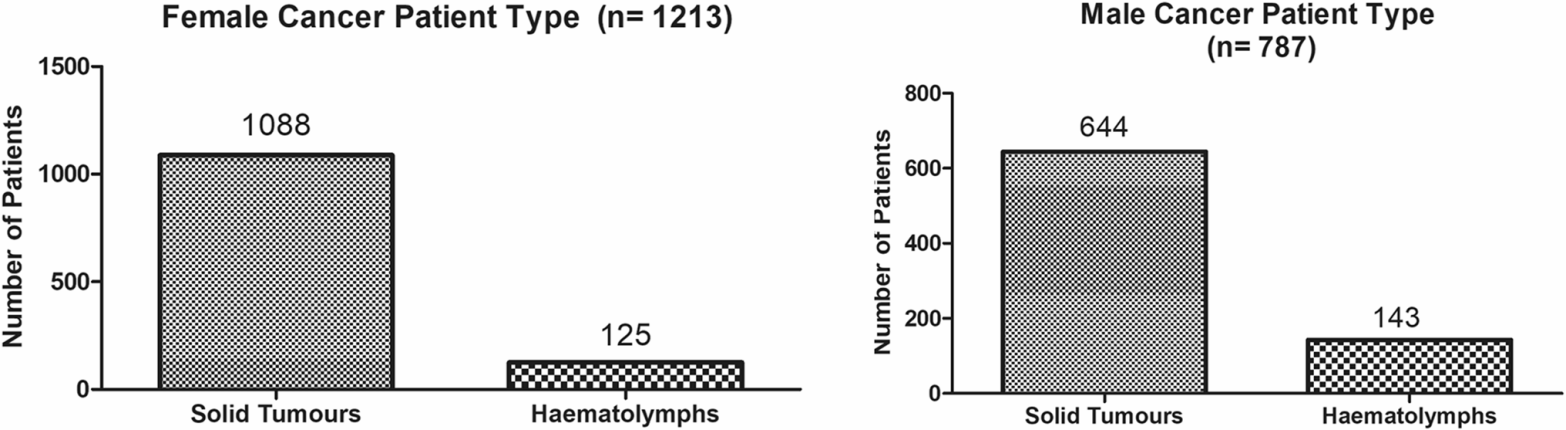
Graph showing type of cancer in female and male patients (ANOVA-Dunnett’s Test, P < 0.05).

**Figure 5:**
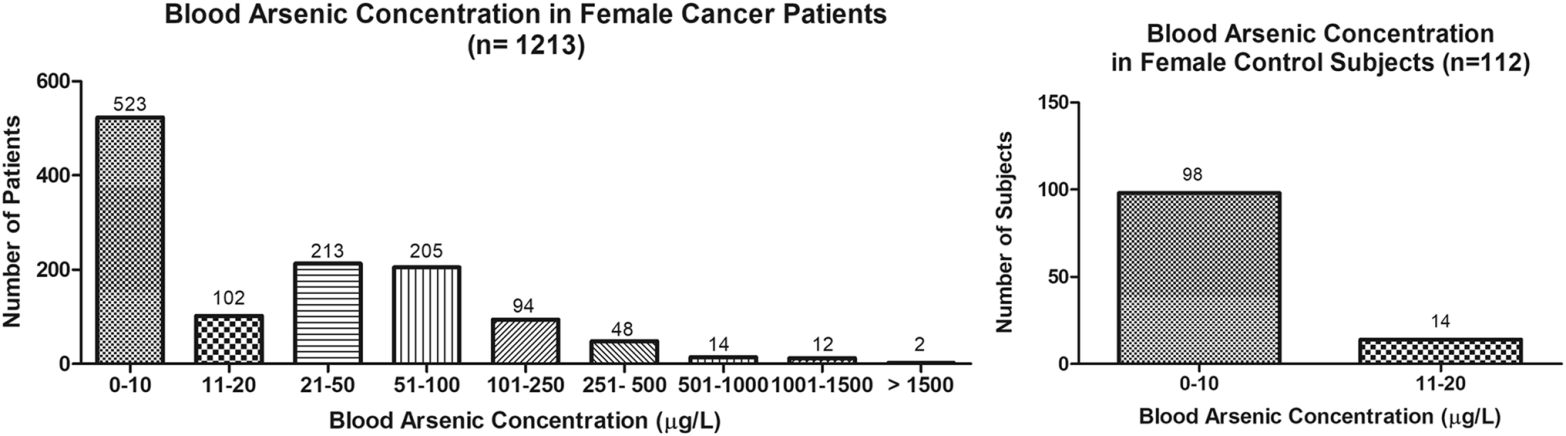
Arsenic concentration in blood samples of female patients were analyzed through GF-AAS (ANOVA-Dunnett’s Test, P < 0.05).

**Figure 6:**
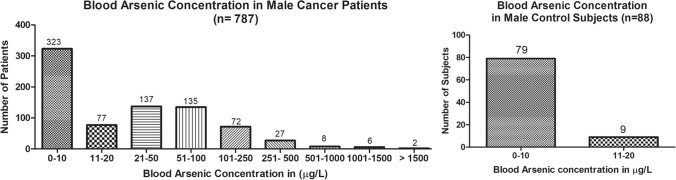
Arsenic concentration in blood samples of male patients were analyzed through GF-AAS (ANOVA-Dunnett’s Test, P < 0.05).

**Figure 7:**
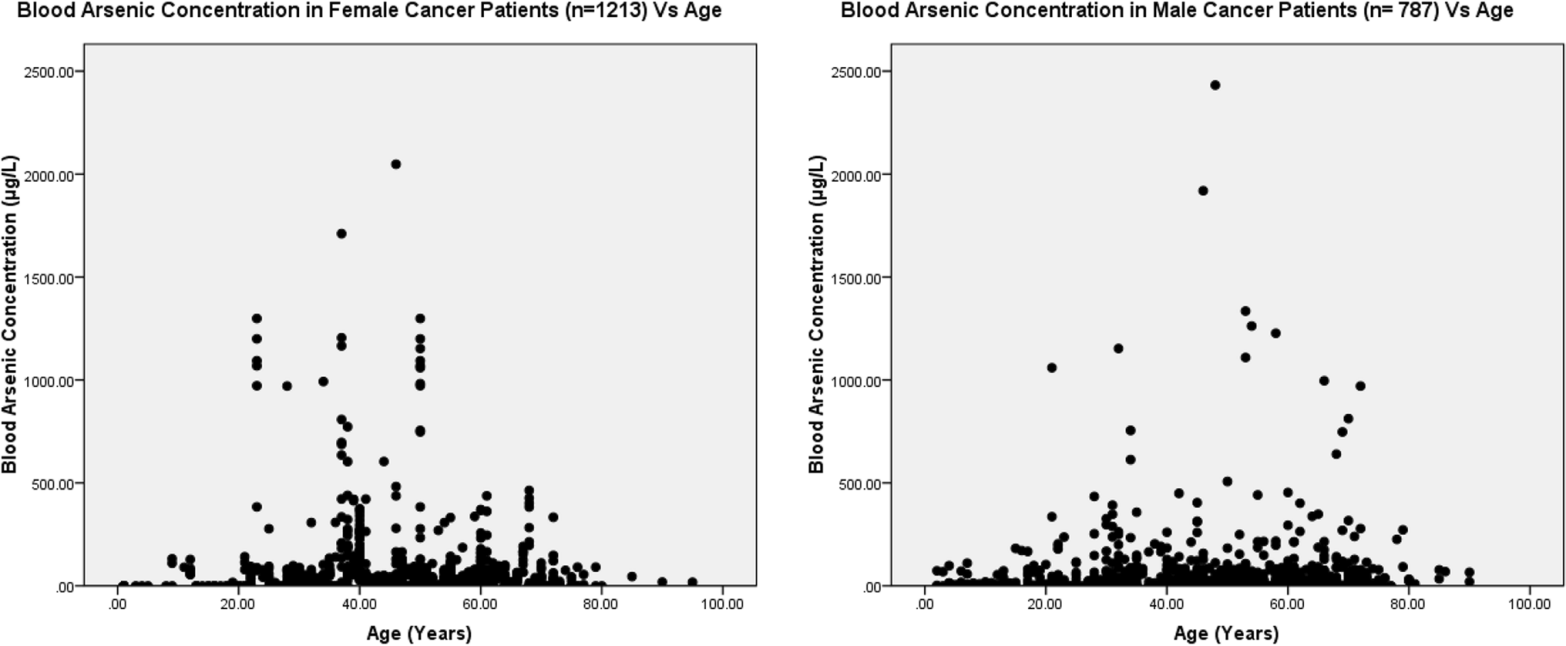
The correlation coefficient between blood arsenic levels and age of the female (n = 1213) (r = 0.005 and P < 0.05) and male (n = 787) cancer patients (in years) (r = 0.003 and P < 0.05).

**Figure 8:**
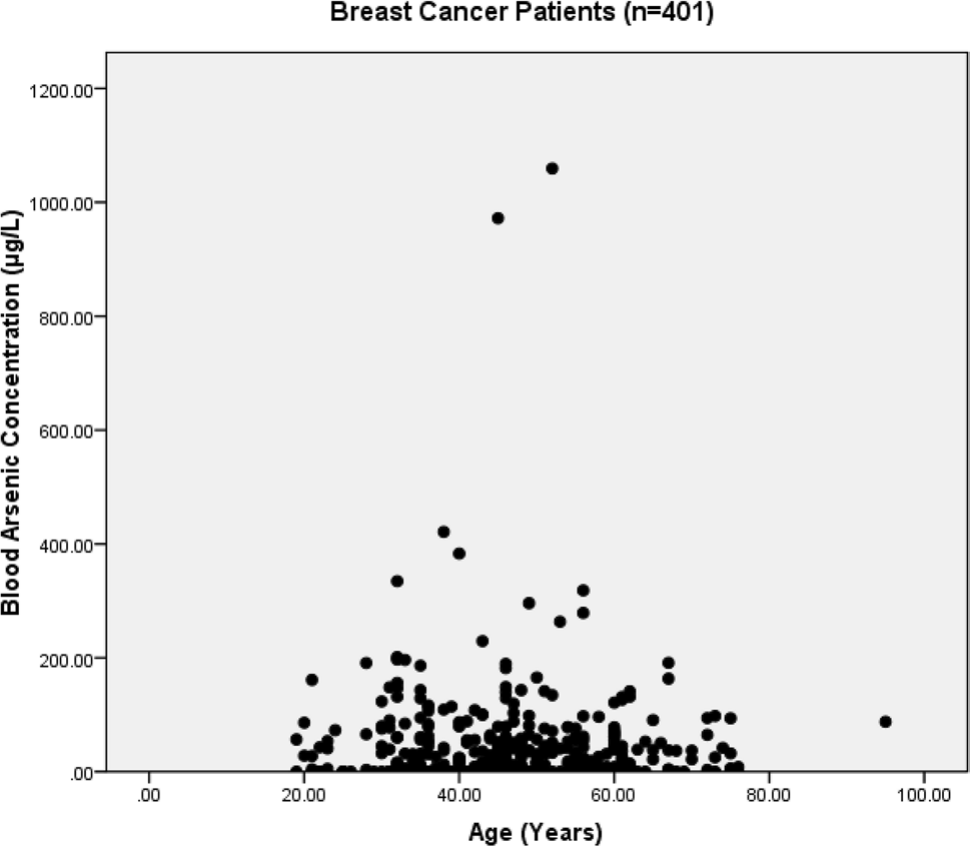
The correlation coefficient between blood arsenic levels and age of the female breast cancer patients (P < 0.05).

**Figure 9:**
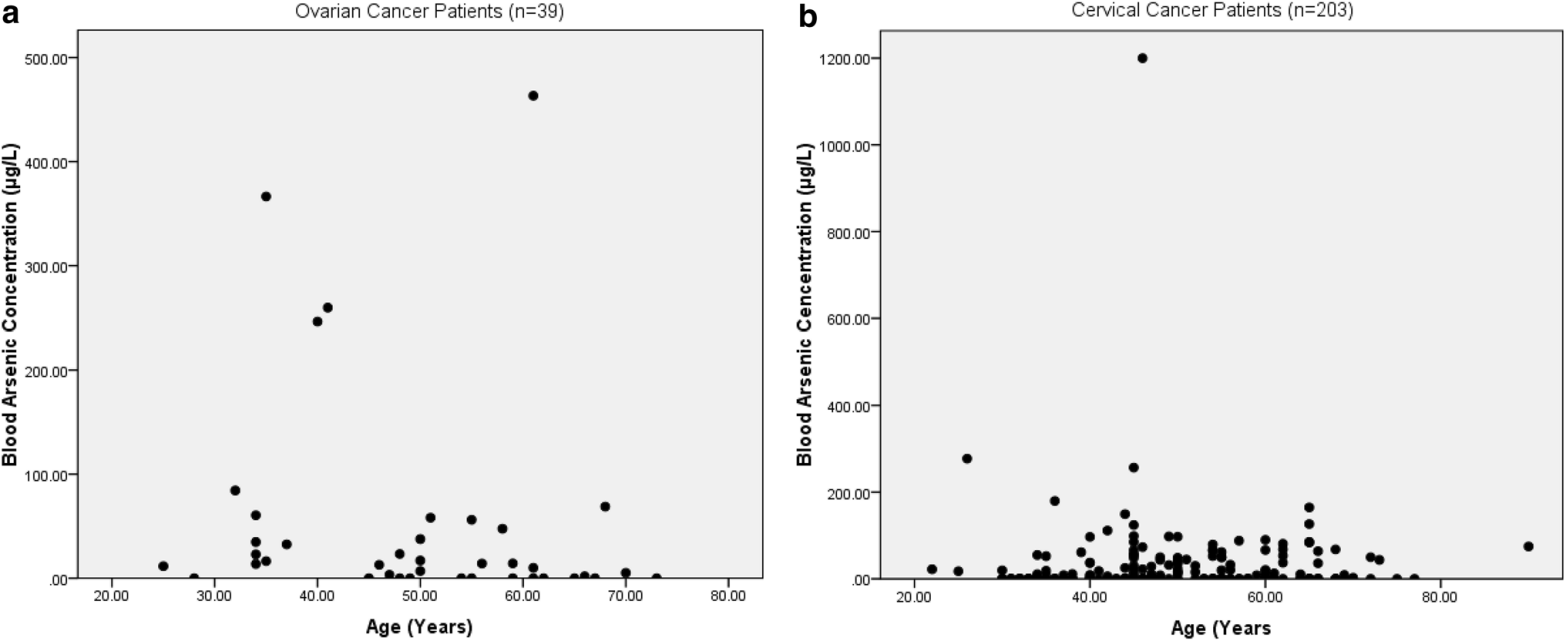
The correlation coefficient between blood arsenic levels and age of the female ovarian and cervical cancer patients (P < 0.05).

**Figure 10:**
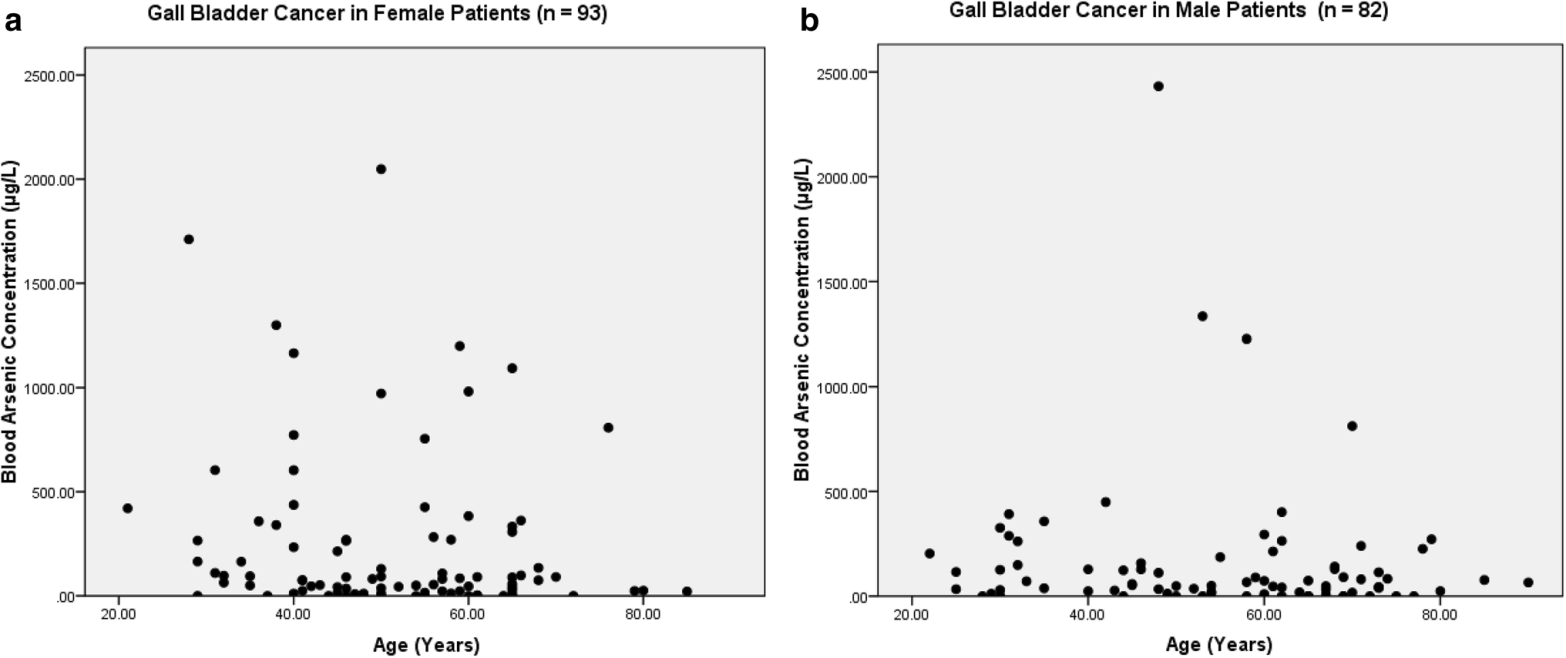
The correlation coefficient between blood arsenic levels and age of the female vs male gallbladder cancer patients (P < 0.05).

**Figure 11:**
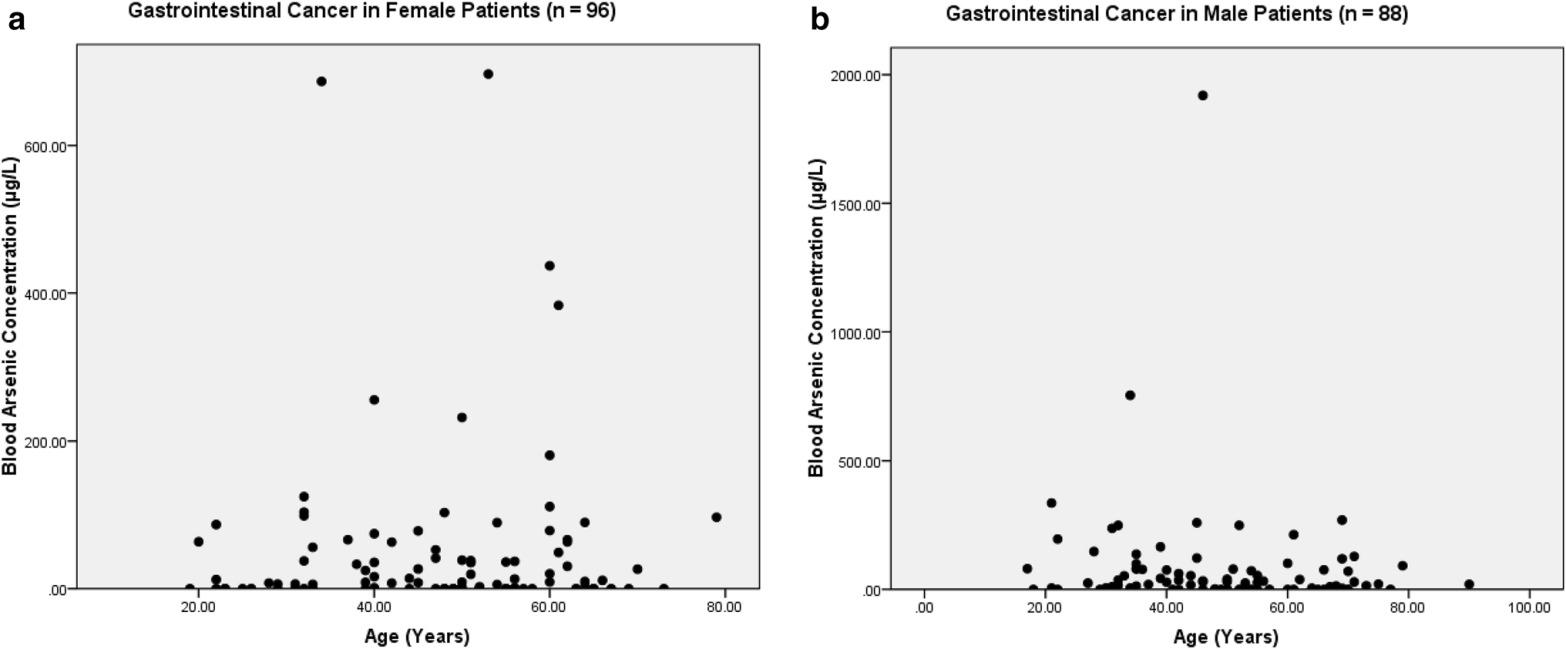
The correlation coefficient between blood arsenic levels and age of the female vs male gastrointestinal cancer patients (P < 0.05).

**Figure 12:**
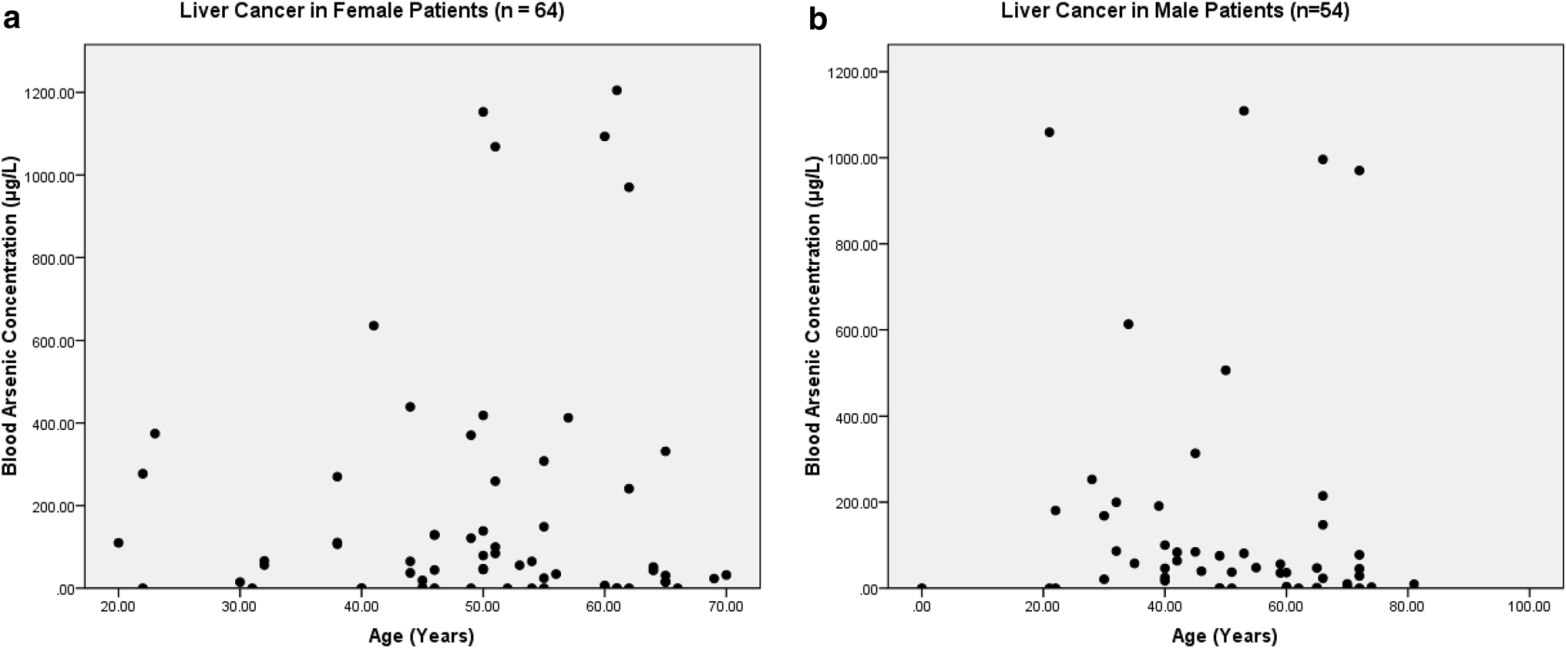
The correlation coefficient between blood arsenic levels and age of the female vs male liver cancer patients (P < 0.05).

**Figure 13:**
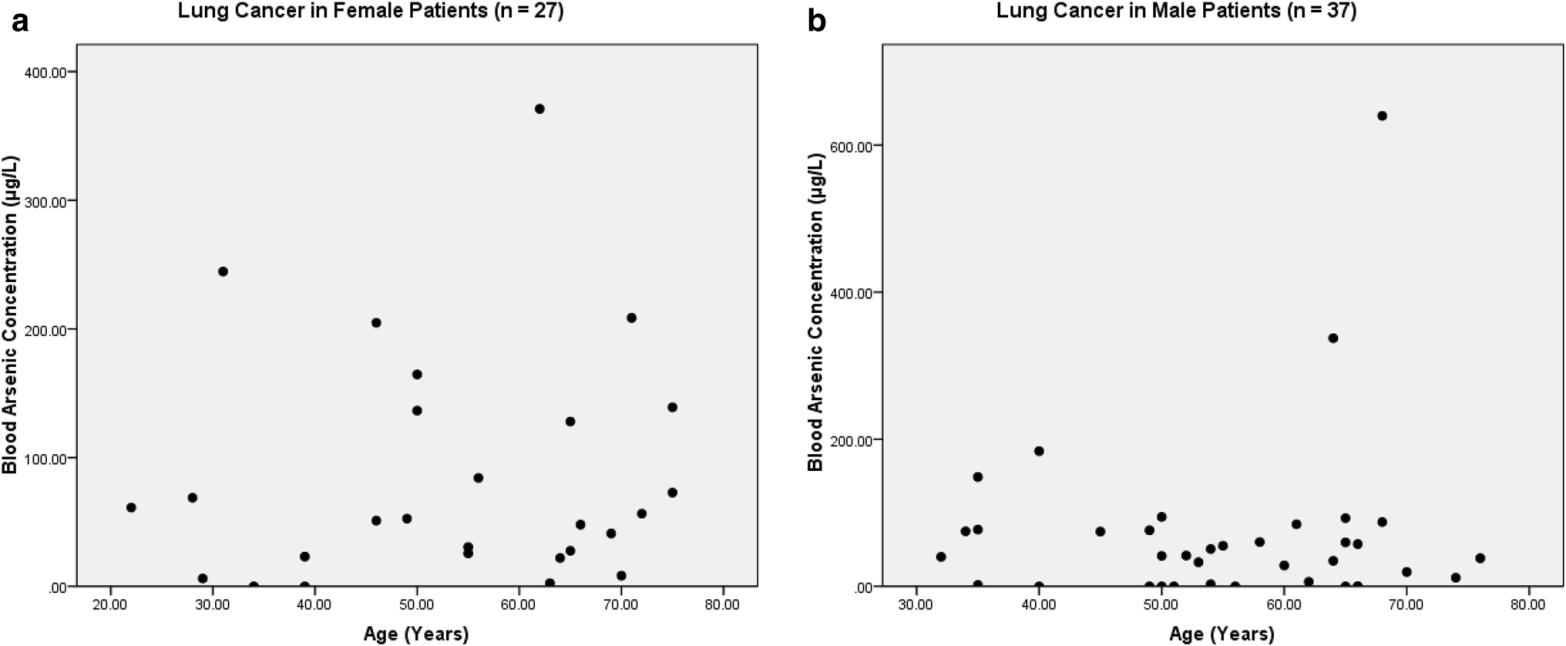
The correlation coefficient between blood arsenic levels and age of the female vs male lung cancer patients (P < 0.05).

**Figure 14:**
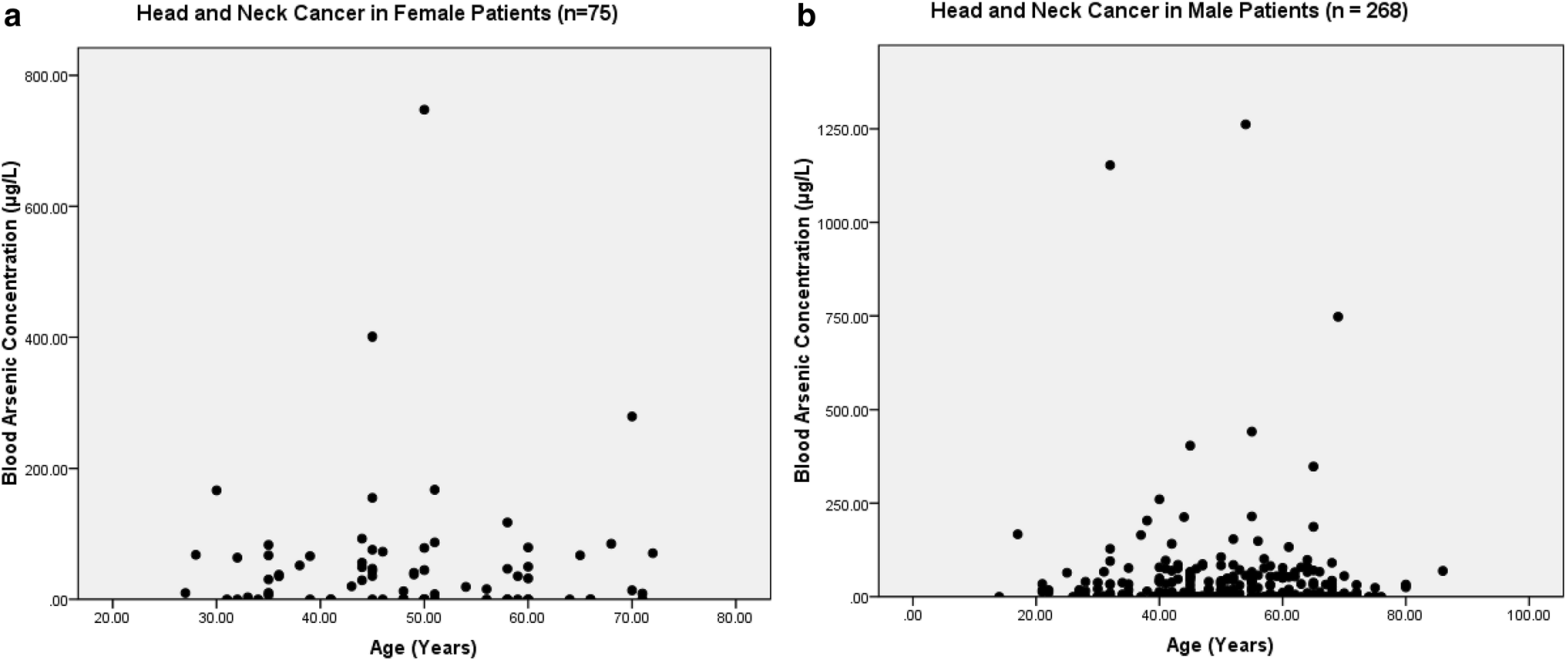
The correlation coefficient between blood arsenic levels and age of the female vs male head and neck cancer patients (P < 0.05).

**Figure 15:**
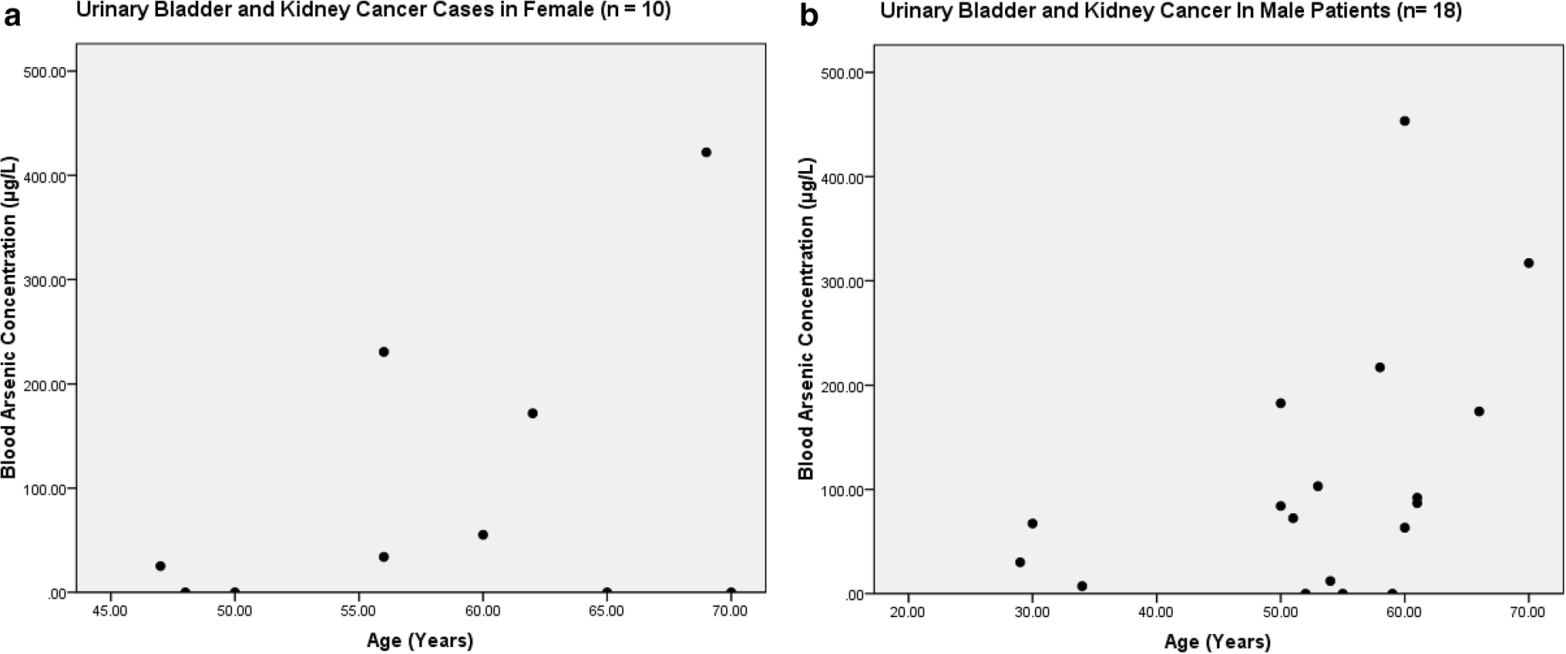
The correlation coefficient between blood arsenic levels and age of the female vs male urinary bladder and kidney cancer patients (P < 0.05).

**Figure 16:**
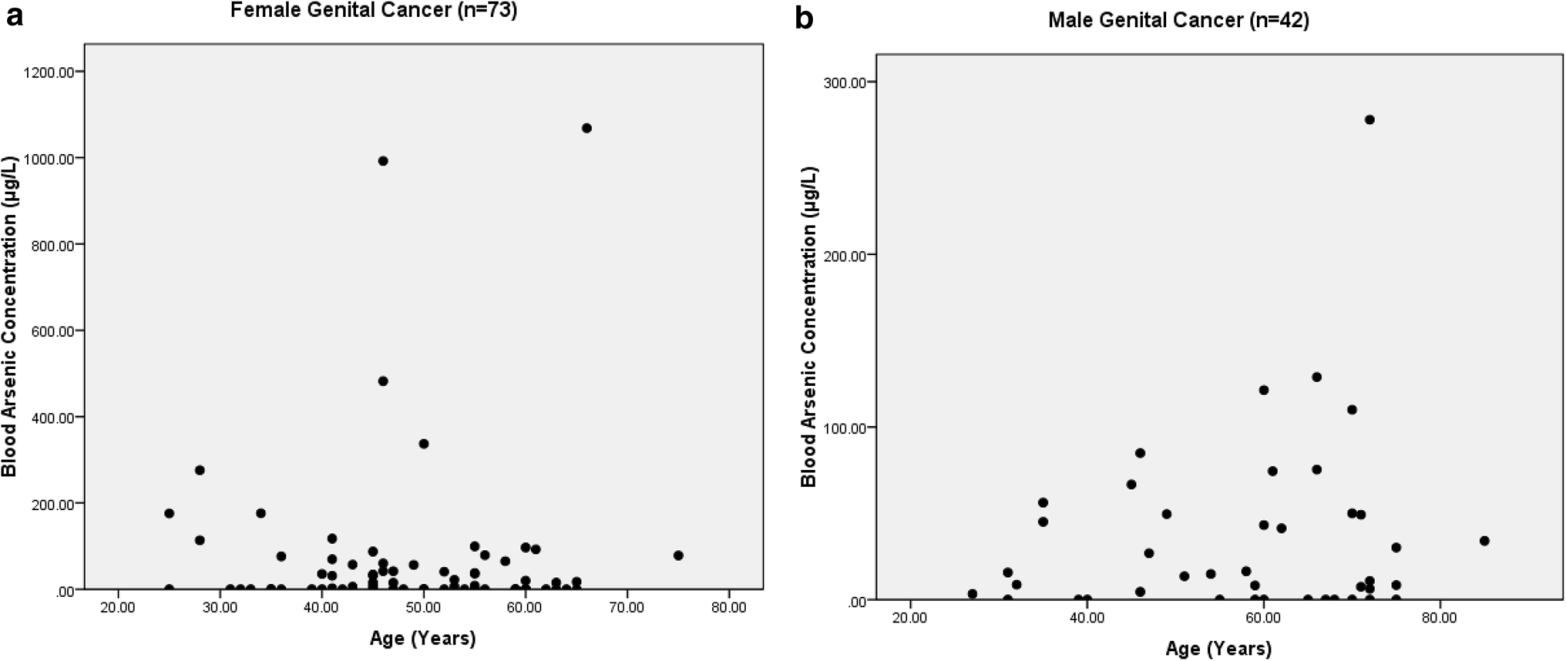
The correlation coefficient between blood arsenic levels and age of the female vs male genital cancer patients (P < 0.05).

**Figure 17:**
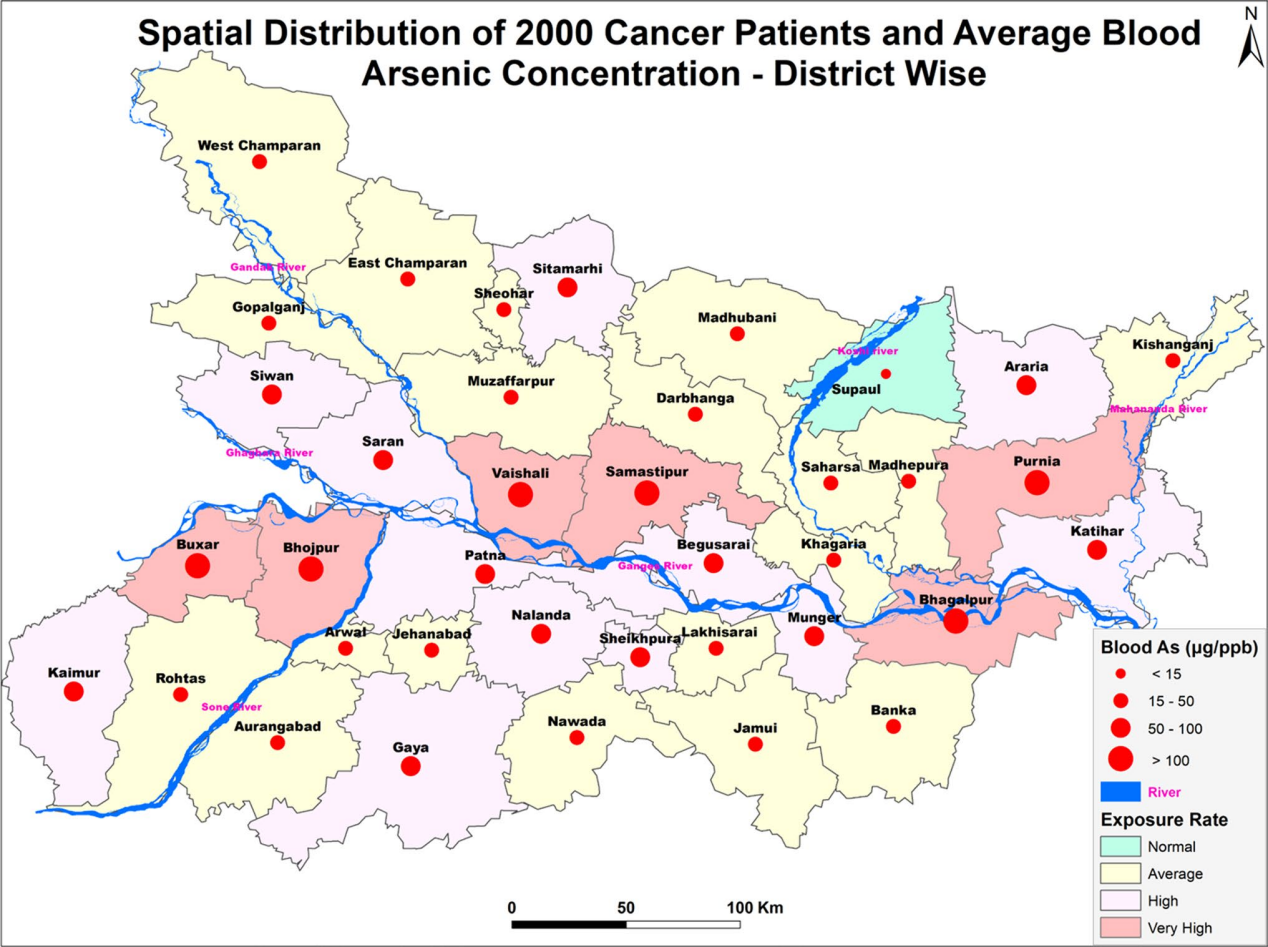
Geospatial Cancer distribution Map of 2000 Cancer patients with Average Blood Arsenic concentration—district wise [Base map extracted from OpenStreetMap—(http://download.geofabrik.de/asia/india.html) using ArcMap10.5.1].

**Figure 18:**
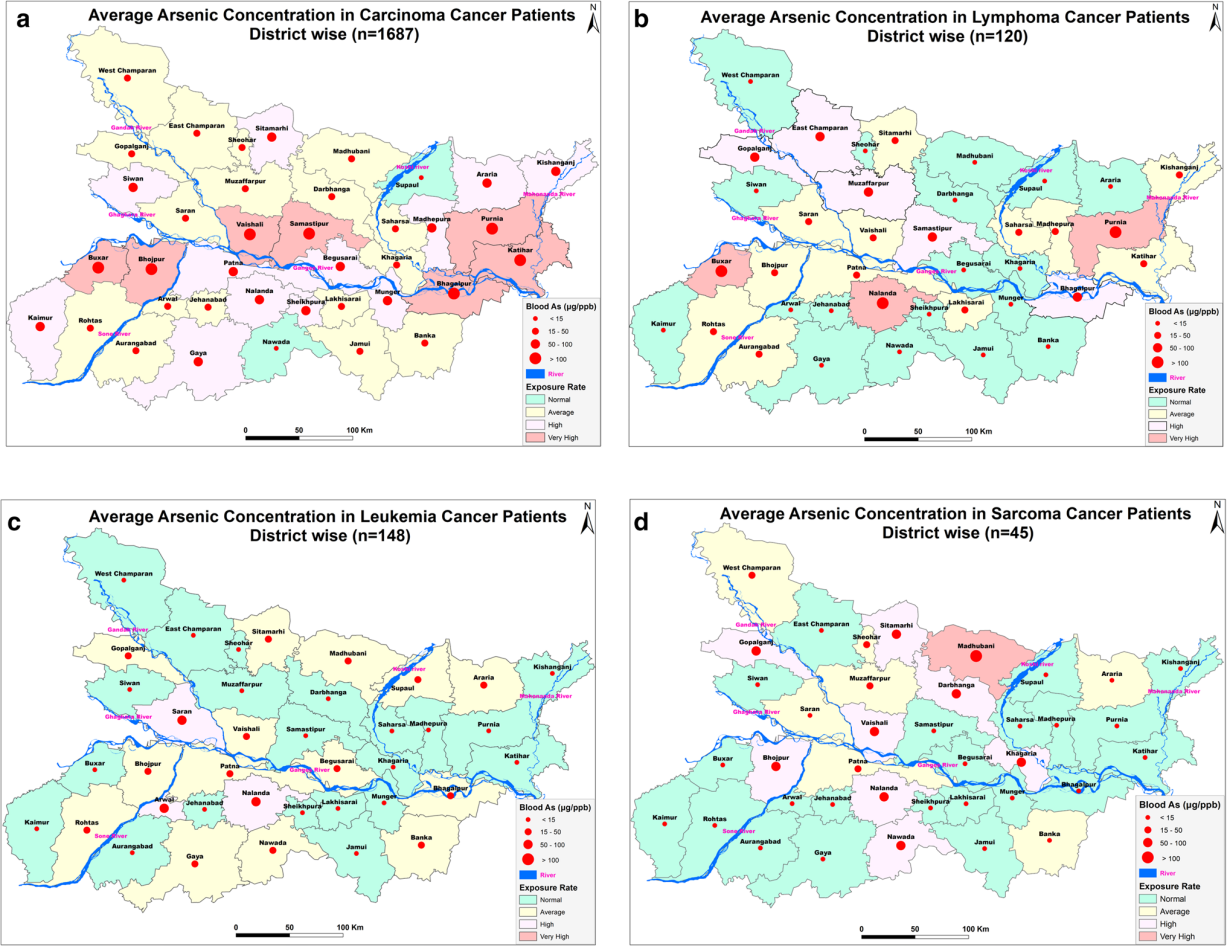
(**A**) Geospatial distribution of Carcinoma Cancer patients with Average Blood Arsenic Concentration [Base map extracted from OpenStreetMap—(http://download.geofabrik.de/asia/india.html) using ArcMap10.5.1]. (**B**) Geospatial distribution of Lymphoma Cancer patients with Average Blood Arsenic Concentration [Base map extracted from OpenStreetMap—(http://download.geofabrik.de/asia/india.html) using ArcMap10.5.1]. (**C**) Geospatial distribution of Leukemia Cancer patients with Average Blood Arsenic Concentration. [Base map extracted from OpenStreetMap—(http://download.geofabrik.de/asia/india.html) using ArcMap10.5.1]. (**D**) Geospatial distribution of Sarcoma Cancer patients with Average Blood Arsenic Concentration. [Base map extracted from OpenStreetMap—(http://download.geofabrik.de/asia/india.html) using ArcMap10.5.1].

**Figure 19:**
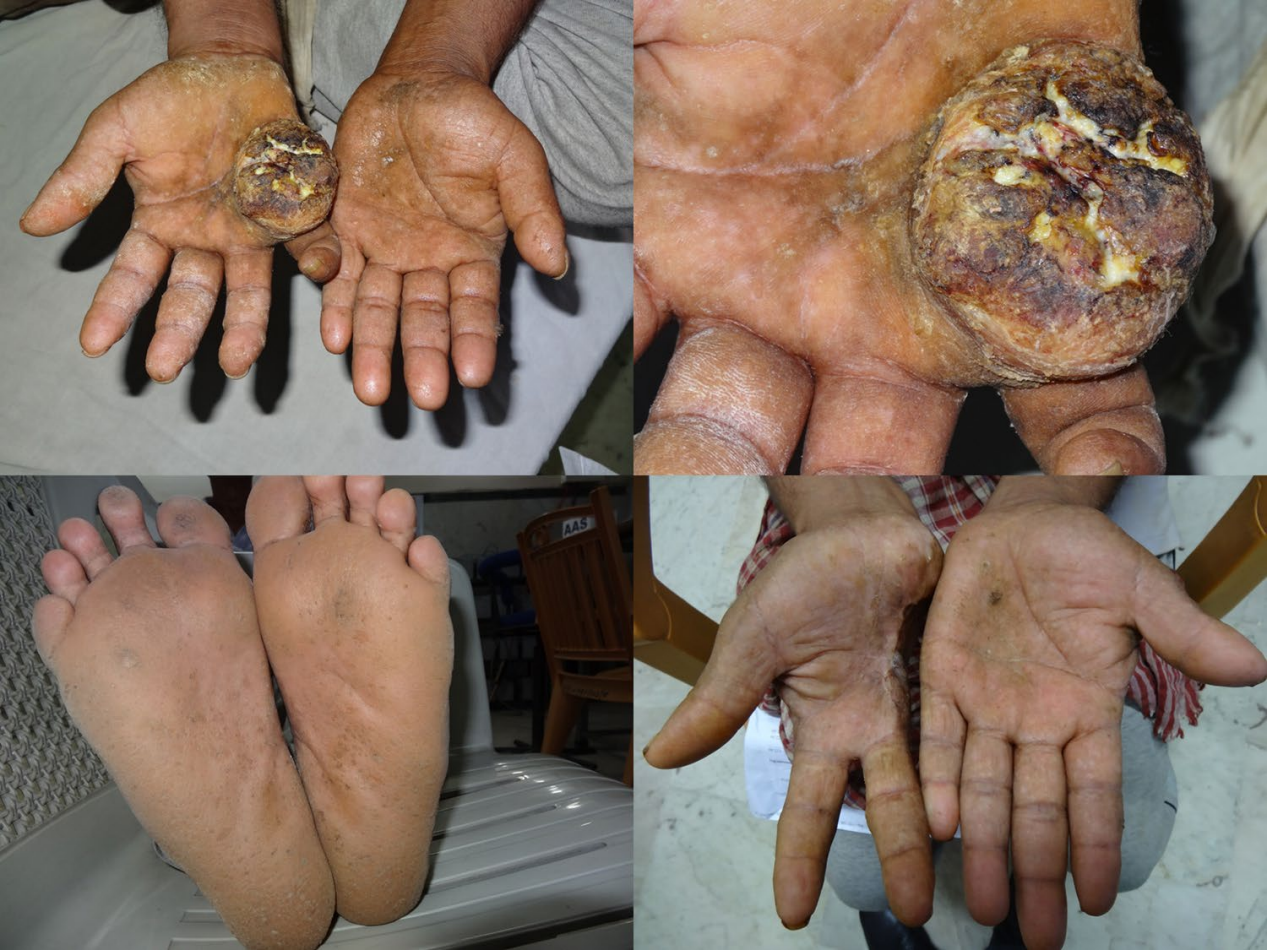
Showing a cancer patient with skin cancer (squamous cell of carcinoma) in his palm with typical arsenicosis symptoms in sole and palm.

1. **Gender wise—Cancer patients vs control subjects:** Total 2000 blood samples of patients were analysed, out of which n = 1213 patients were cancer female patients while n = 787 subjects were male cancer patients. In the total studied 200 control subjects, n = 112 were the female subjects, while n = 88 were the male subjects (Fig. 1).
2. **Age wise in female cancer patients:** In total n = 1213 female cancer patients, the maximum cancer incidences were observed in the patient’s age group between 31–70 years. In the control female subjects n = 112, the maximum studied groups were between 21–70 years of age group. The age group between 21–30 had the highest number of the studied subjects n = 56 (Fig. 2).
3. **Age wise in male cancer patients:** In total n = 787 male cancer patients, the maximum cancer incidences were observed in the patient’s age group between 21–70 years. In the control male subjects n = 88, the maximum studied groups were between 21–70 years of age group. The age group between 21–30 had the highest number of the studied subjects n = 44 (Fig. 3).
4. **Type of cancer in female and male cancer patients:** Out of n = 1213 females, n = 1088 cases were of solid tumours while, n = 125 females were of haematolymph cases while, out of n = 787 males, n = 644 cases were of solid tumours while, n = 143 males represented haematolymph cases (Fig. 4).
5. **Blood arsenic concentration in female cancer patient’s vs female control subjects:** Out of total n = 2000 blood samples of cancer patients analysed, n = 1213 (60%) blood samples were of female patients and the maximum arsenic concentration in their blood sample reported was 2048 μg/L. The minimum value of arsenic in blood was observed to be between 0-10 μg/L. In the present data, n = 523 (43.11%) blood samples were in the minimum range between 0–10 μg/L, out of which n = 395 patients had their blood arsenic concentration values less than 1.0 μg/L. The n = 102 subjects had the blood arsenic concentration between the range 11–20 μg/L. The rest n = 588 (48.47%) patients had the blood arsenic concentration more than the minimum range. In the control female subjects n = 112, maximum subject n = 98 (87.5%) had the blood arsenic concentration with in the minimum range 0–10 μg/L, the rest n = 14 (12.5%) had mild blood arsenic concentration in their bloods in the range between 11–20 μg/L. The maximum arsenic concentration in their blood sample reported was 19.4 μg/L (Fig. 5).
6. **Blood arsenic concentration in male cancer patient’s vs male control subjects:** Out of total n = 2000 blood samples of cancer patients analysed, n = 787 (40%) blood samples were of male patients and the maximum arsenic concentration in their blood sample reported was 2432 μg/L. The minimum value of arsenic in blood was observed to be between 0–10 μg/L. In the present data, n = 323 (41.04%) blood samples were in the minimum range between 0–10 μg/L, out of which n = 236 patients had their blood arsenic concentration values less than 1.0 μg/L. The n = 77 subjects had the blood arsenic concentration in the range 11–20 μg/L. The rest n = 387 (49.17%) patients had the blood arsenic concentration more than the minimum range. In the control male subjects n = 88, maximum subject n = 79 (89.7%) had the blood arsenic concentration with in the minimum range 0–10 μg/L, the rest n = 9 (10.2%) had mild blood arsenic concentration in their bloods in the range between 11–20 μg/L. The maximum arsenic concentration in their blood sample reported was 19.6 μg/L (Fig. 6).
7. **Correlation coefficient between blood arsenic levels and age of the female and male cancer patients:** The study showed significant increase in blood arsenic levels in the female cancer patients (r = 0.005 and P < 0.05) and male cancer patients (r = 0.003 and P < 0.05) (Fig. 7).
8. **Correlation coefficient between blood arsenic levels and age of the female breast cancer patients:** The study showed significant increase in the blood arsenic levels in the female breast cancer patients n = 401; (P < 0.05) (Fig. 8).
9. **Correlation coefficient between blood arsenic levels and age of the female ovarian and cervical cancer patients:** The study showed significant increase in the blood arsenic levels in the female ovarian (n = 39) and cervical (n = 203) cancer patients (P < 0.05) (Fig. 9).
10. **Correlation coefficient between blood arsenic levels and age of the Gall bladder Cancer—female vs male patients:** The study showed significant increase in the blood arsenic levels in the female (n = 93) vs male (n = 82) gallbladder cancer patients; (P < 0.05) (Fig. 10).
11. **Correlation coefficient between blood arsenic levels and age of the Gastrointestinal Cancer—female vs male patients:** The study showed significant increase in the blood arsenic levels in the female (n = 96) vs male (n = 88) gastrointestinal cancer patients (P < 0.05). This includes total gastrointestinal cancer cases (n = 184). Out of which the cancer of stomach was (n = 90), esophagus (n = 18), iliac (n = 09), colon (n = 21), rectum (n = 29) and anus (n = 17) (Fig. 11).
12. **Correlation coefficient between blood arsenic levels and age of the Liver Cancer—female vs male patients:** The study showed significant increase in the blood arsenic levels in the female (n = 64) vs male (n = 54) liver cancer patients; (P < 0.05) (Fig. 12).
13. **Correlation coefficient between blood arsenic levels and age of the Lung Cancer—female vs male patients:** The study showed significant increase in the blood arsenic levels in the female (n = 27) vs male (n = 37) lung cancer patients; (P < 0.05) (Fig. 13).
14. **Correlation coefficient between blood arsenic levels and age of the Head and Neck Cancer—female vs male patients:** The study showed significant increase in the blood arsenic levels in the female (n = 75) vs male (n = 268) head and neck cancer patients; (P < 0.05) (Fig. 14).
15. **Correlation coefficient between blood arsenic levels and age of the Urinary bladder and Kidney Cancer—female vs male patients:** The study showed significant increase in the blood arsenic levels in the female (n = 10) vs male (n = 18) urinary bladder and kidney cancer patients; (P < 0.05) (Fig. 15).
16. **Correlation coefficient between blood arsenic levels and age of the female and male genital cancer patients:** The study showed significant increase in the blood arsenic levels in the female (n = 73) genital cancer patients (P < 0.05). The female genital cancer cases included cancer of vagina, vault and uterus. The study also showed significant increase in the blood arsenic levels in the male (n = 42) genital cancer patients (P < 0.05). The genital cancer cases included cancer of prostate, testis, penis, seminal vesicle and scrotum (Fig. 16).
17. **Geospatial Cancer distribution Map of 2000 Cancer patients with Average Blood Arsenic concentration—district wise:** The district wise map shows the geospatial distribution of 2000 cancer patients with average blood arsenic concentration (Fig. 17).
18. **Cancer types:** The cancer types have been primarily categorised into 04 major parts—leukaemia’s, lymphomas, sarcomas and carcinomas. They have been further categorised into 18 subtypes. All the data have been correlated with blood arsenic concentration and their age. In carcinomas, the maximum number observed were of Breast cancer (n = 401), Head and Neck cancer (n = 343), Cervical cancer (n = 203), Gastro intestinal cancer (n = 184), Gall bladder cancer (n = 175), Liver cancer (n = 118) and the remaining types had their numbers less than 100. (Table 1).
19. **Geospatial distribution of Cancer types:** The maps show the district wise geospatial distribution of cancer types with average blood arsenic concentration. The district wise distribution with average blood arsenic is very significant with the level of arsenic exposure in Carcinoma (Fig. 18A), Lymphoma (Fig. 18B), Leukemia (Fig. 18C), Sarcoma Fig. 18D).
20. **Skin cancer patient with arsenicosis symptoms:** The studied patient was having skin cancer—squamous cell of carcinoma, drinking arsenic contaminated water of 322 μg/L and his blood arsenic concentration was 86.4 μg/L (Fig. 19).

**Table 1:** Cancer type details of patients.

Type of cancer	Total number of cancer cases	Mean ± SE blood arsenic concentration (µg/L)	Maximum blood arsenic concentration (µg/L)	Minimum blood arsenic concentration (µg/L)	Lower 95% CI of mean	Mean ± SE subject age (years)	Maximum subject age (years)	Minimum subject age (years)	Lower 95% CI of mean
1. Leukemias	148	25.92 ± 3.939	434.00	0	18.138	26.14 ± 1.549	76	01	23.073
2. Lymphomas	120	52.57 ± 12.24	1298.60	0	28.333	39.50 ± 1.492	77	04	36.545
3. Sarcomas	45	46.28 ± 10.29	347.04	0	25.538	29.91 ± 2.378	70	07	25.118
4. Carcinomas	1687	73.81 ± 4.421	2432	0	65.139	49.00 ± 0.341	95	01	48.333
5. Breast cancer	401	46.86 ± 4.553	1059.40	0	37.912	46.55 ± 0.621	95	19	45.329
6. Cervical cancer	203	29.91 ± 6.490	1199.80	0	17.111	49.25 ± 0.832	90	22	47.610
7. Ovarian cancer	39	51.00 ± 16.55	463.24	0	17.492	49.82 ± 2.041	73	25	84.512
8. Female genital cancer	73	70.23 ± 21.21	1068.40	0	27.947	48.60 ± 1.264	75	25	46.082
9. Lung cancer	64	75.52 ± 13.15	639.80	0	49.249	54.42 ± 1.706	76	22	51.012
10. Gall bladder cancer	175	201.7 ± 27.86	2432	0	146.684	53.06 ± 1.114	90	21	50.857
11. Liver cancer	118	169.9 ± 26.78	1205	0	116.843	50.58 ± 1.288	81	20	48.0337
12. Kidney and urinary bladder cancer	28	103.7 ± 23.87	453.04	0	54.713	54.86 ± 2.008	70	29	50.7362
13. Head and neck cancer	343	46.46 ± 6.485	1262	0	33.71	50.56 ± 0.725	86	14	49.13
14. Stomach cancer (GI)	90	98.57 ± 25.25	1919.2	0	48.3935	50.67 ± 1.326	79	22	48.031
15. Esophageal cancer (GI)	18	11.68 ± 4.912	76.0	0	1.314	56.11 ± 3.615	77	18	48.48
16. Iliac cancer (GI)	09	38.18 ± 28.45	259.2	0	-27.43	41.22 ± 3.647	56	25	32.81
17. Colon cancer (GI)	21	48.56 ± 16.49	232.0	0	14.15	40.95 ± 3.801	90	19	33.02
18. Rectum cancer (GI)	29	48.48 ± 16.11	335.8	0	15.48	43.21 ± 2.788	71	21	37.50
19. Anus cancer (GI)	17	39.76 ± 15.69	249.0	0	6.499	45.12 ± 4.403	75	17	35.78
20. Male genital cancer	42	35.32 ± 8.047	278	0	19.064	57.71 ± 2.301	85	27	53.068
21. Skin cancer	05	50.62 ± 20.58	88.68	0	-6.533	56.80 ± 4.91	72	42	43.16
22. Others (glioblastoma and Wilm’s tumour)	07	41.60 ± 15.70	181.07	0	7.037	19.75 ± 6.507	70	01	5.429

Data are represented as mean ± standard error using (ANOVA—Dunnett’s test, P < 0.05).

### Geological perspective

A detail assessment of incidence of arsenic in groundwater has been attempted in the middle Ganga plain in the Bhojpur and Patna districts of Bihar to study the distribution pattern and controlling factors of its occurrence. Groundwater samples were tested with field kit for arsenic, 3D surface maps were prepared at different depth levels and a positive correlation of geomorphology and depth with incidence of arsenic was worked out. It was observed that while the older alluvium surface in the area is free from hazardous incidence of arsenic, the older/present day flood plain surface has several localized pockets of higher incidence of arsenic (50 to  > 500 μg/L). There is a specific depth control observed where the aquifer within 12–75 m depth range is yielding arsenic. Since, older alluvium (Peninsular origin) is free from hazardous incidences of arsenic in ground water, the source of arsenic contamination in the ground water appears to be associated with the holocene sediments of the Himalayan provenance brought down by the river Ganga and its tributaries of extra peninsular (Himalayan) origin^31^.

### Ethics approval and consent to participate

Ethical approval was obtained from the Institutional Ethics Committee (IEC) of Mahavir Cancer Sansthan and Research Centre with IEC No. MCS/Research/2015-16/2716, dated 08/01/2016. Furthermore, it is also certified that the informed consent was taken from the individuals who voluntarily participated in this study while the minor’s parents provided us the informed consent for this particular study.

## Discussion

In the human metabolic system, inorganic arsenic through drinking water reaches the blood through gastrointestinal tract. It is easily converted into organic form which in excess is primarily eliminated through urine. In the blood it remains for 2–6 hours and is mostly eliminated through the renal system^32,33^. It is also deposited in the keratin of skin, hair and nails and alters the epidermal keratinocytes causing keratosis, melanosis, rain drop pigmentation or other skin manifestations^34^. The arsenic adversely effects the epidermal system, the vascular system and the nervous system of human beings. The acute poisoning causes vomiting, diarrhea, abdominal pain, general body weakness, vertigo, nausea, muscle cramps etc. The long duration arsenic exposure causes skin manifestations like keratosis, melanosis in sole and palm along with rain drop pigmentation all over the body. Further, it also causes peripheral neuropathy, renal failure, gastrointestinal disruption, hypertension, diabetes, conjunctivitis, anaemia, loss of appetite, breathlessness, mental disability, hormonal imbalances, suppression of bone marrow and cardiovascular diseases^35–44^. The trivalent arsenic is more toxic than the pentavalent arsenic hence is known to be a carcinogen^4,10,45,46^. There are mainly three ways by which humans are exposed to arsenic—drinking arsenic contaminated groundwater, food prepared with the arsenic contaminated water and food crops irrigated with high arsenic contaminated groundwater. This causes entry of arsenic into human body through various routes causing life threatening disease like cancer of the skin, bladder, lungs, kidney, liver, and prostate^47^. There is adequate evidence which states that arsenic causes carcinogenicity in humans and the International Agency for Research on cancer has classified arsenic as Category-I carcinogen^4^. The skin cancer, Bowen’s disease and squamous cell carcinoma are very common in arsenic exposed population^4,48–51^. In recent studies, it has been found that arsenic is causing reproductive health hazards as cases of spontaneous abortion, stillbirth and preterm birth. The pregnant women who are continuously drinking arsenic contaminated water are also exposing their foetus through placenta causing severe health problem for the child after birth^52,53^.

During the course of this study, we have observed very high arsenic concentration in ground water samples (1929 μg/L) in Buxar district of Bihar. From the same household, we have also observed the blood arsenic concentration in a subject as 664.6 μg/L which is the highest reported case in the state of Bihar showing typical symptoms of arsenicosis^12^. Similar studies have been reported by many other researchers^54–60^.

The blood arsenic concentration in the exposed population has been rarely studied, since it is thought to be very weak biomarker. Hence, there had been no benchmark established for the blood arsenic concentration for humans. The present comprehensive study carried out is the world’s first study which deciphers the association between arsenic and cancer through blood arsenic study. In the present study, there was significantly high arsenic concentration in the blood samples of cancer patients especially in the females in comparison to male. This denotes that there is some pathway, which makes the arsenic exposed population more vulnerable, which subsequently due to sustained exposure gets converted into a life-threatening disease like cancer. Secondly, the significant levels of arsenic concentration have a distinct correlation with the incidences of cancer patients. The cross-sectional design also correlates that the non-cancer subjects (control subjects) hardly had any arsenic exposure, as n = 135 had zero arsenic concentration, while n = 42 between 1–10 μg/L of minimum range. However, n = 23 had very mild blood arsenic concentration levels between 10–20 μg/L. This explains our observations made as a part of study. Moreover, out of 2000 studied cancer cases, in the n = 1154 (57.7%) cancer cases, the blood arsenic concentration was found to be more than the minimum range (0–10 μg/L) and while n = 846 (42.3%) were in the minimum range. The coefficient correlation also is directly proportional to higher the age of the subject, more is the blood arsenic concentration. Thus, arsenic is first weakening the immune system and then causing the health ailments like cancer. The geospatial distribution of studied cancer patients with blood arsenic concentration in their blood also correlates that the disease burden is very high in the Gangetic basin of the state where the arsenic contamination in ground water is also relatively very high^61–63^. In a recent study carried out by our team in Patna district, Bihar, cancer mapping in Gyaspur Mahaji village has been extensively done to establish the relation and the study strongly correlates the association^64^.

Various studies have deciphered the molecular pathway of arsenic causing cancer in the subjects from arsenic exposed area. In squamous cell carcinoma of skin, arsenic binds with the receptors and disrupts the signal transduction pathways. The arsenic affinity to bind with sulfhydryl (SH) groups causes release of Reactive Oxygen Species (ROS) which leads to cellular toxicity and metabolic dysfunction^65,66^. The interaction of arsenic with thiol groups is associated with 200 known human proteins. These interactions cause production of ROS which leads to activation of oncogenes, upregulation of inflammatory pathways and inhibition of the function of tumour suppressor genes^41,67–71^.

In a recent study by some researchers, it is speculated that arsenic activates the cell proliferation through Canonical Hippo Signaling pathway which causes various types of malignancies including skin cancer^72,73^. Furthermore, arsenic upregulates the various components of Hippo signaling including mammalian STE20-like kinase STE20-like kinase 1/2 (Mst1), Salvador homolog 1 (Sav1), large tumour suppressor kinase 1/2 (LATS1) and Mps one binder kinase activator-like 1A (MOB1). In the epithelial cell proliferation Yes-associated protein (YAP) is a responsible component is dephosphorylated by arsenic causes the control over tight/adherens junctions of the epithelium^74,75^. In the recent times, various cancer types and its cause due to arsenic has been established say in case of bladder cancer^76–82^, for lung, kidney and laryngeal cancer^83–86^. In a study conducted in Brazil showed significant levels of blood arsenic in maternal chord blood with limit above 3.30 µg/L^87^. Study in another city in Brazil showed significant blood arsenic reference value as 9.87 µg/L^88^. While in a study carried out in 32 children in Yucatan, Mexico showed blood arsenic levels above 10 µg/L in 37% of the samples^89^. In a similar study conducted on 120 arsenic exposed residents (76 breast cancer cases) of Camarca Lagunera, Mexico showed the expression of Yes- Associated Protein (YAP), a tumour suppressor protein along with apoptosis inhibitor was measured. The result showed low percentage of YAP expression denotes abnormal expression of YAP in arsenic exposed breast cancer patients^90,91^.

Our institute (MCSRC) has registered more than 15,000 cancer cases in year 2019. The epidemiological data showed that most of the cancer cases reported were from the cities or towns which are located near the river Ganga. The most incidences of cancer cases were from the districts—Buxar, Bhojpur, Saran, Patna, Vaishali, Samastipur, Munger, Begusarai Bhagalpur etc. Various studies have reported that arsenic in the form of arsenopyrite load has reached these river basins in the form of silt from great Himalayas and has caused geogenic changes in the sediments and ground water causing health hazards to the exposed population^92,93^. It is evident from many studies that consumption of arsenic contaminated groundwater for drinking purposes and contaminated food has caused health related issues in the population in long duration exposure finally leading to cancer disease. Our epidemiological data also suggests that the districts located near the course of Himalayan bound river basins have more incidences in comparison to non-Himalayan river basins. Most common cancer cases recorded were of skin cancer, skin melanoma, lung cancer, bladder cancer, hepatobiliary cancer, renal cell carcinoma, breast cancer, ovarian cancer, endometrium cancer etc. with typical symptoms of arsenicosis denotes that there is a significant correlation with the arsenic. The arsenic contamination in the long duration of exposure is causing the exposed population contract the disease in primary phase and then acquiring second stage of disease, if not cured in time. It is quite possible that arsenic along with other confounding factors could be adding the disease burden. Apart from this, the cancer disease types like carcinomas are more aggressive than the other types like leukemias, lymphomas and sarcomas. The pathways related to cause of cancer in the arsenic exposed population needs further studies for the final validation and establishment of benchmark for blood arsenic concentration in humans.

## Conclusion

The present study demonstrates the high incidence of cancer in arsenic prevalent, Gangetic basin. The study strongly correlates the association between arsenic and cancer incidence in the arsenic exposed population of Bihar in which significantly high arsenic concentration has been observed in the blood samples of cancer patients. Arsenic exposure also correlates with the high incidence of cancer disease burden in the carcinoma type of cancer in comparison to the sarcomas, lymphomas and leukemias type of cancer. This study reiterates the fact that the people living in the Gangetic basin are getting exposed to the continued arsenic toxicity leading to the development of several types of cancers. More systematic study is further required to understand the molecular mechanisms of arsenic toxicity in incidences of cancer and its progression and to establish the correlation by deciphering the signaling pathways for arsenic exposed human cancer. All this effort will eventually lead to the development of improved therapeutic approach.

## Data Availability

The data that support the findings of this study is available from the corresponding author upon reasonable request.
